# Supplementary material for: Prevalence and dynamics of antimicrobial resistance in pioneer and developing Arctic soils
Source: BMC Microbiol. 2025 Jan 27;25:50. doi: 10.1186/s12866-025-03745-7 (PMC11771051; doi:10.1186/s12866-025-03745-7)
Supplement: Supplementary file 1 — Supplementary Material 1 [file 12866_2025_3745_MOESM1_ESM.pdf]

Supplementary information for

Prevalence and dynamics of antimicrobial resistance in pioneer and developing Arctic soils

Shamik Roy[1], Robin A. Dawson[1], James A. Bradley[2,3], Marcela Hernández[1*]

[1]School of Biological Sciences, University of East Anglia, Norwich NR4 7TJ, UK

[2]Aix Marseille Univ, Université de Toulon, CNRS, IRD, MIO, Marseille, France

[3]School of Biological and Behavioural Sciences, Queen Mary University of London, London, UK

*Corresponding author: Marcela Hernández, School of Biological Sciences, University of East Anglia, Norwich NR4 7TJ, UK, marcela.hernandez@uea.ac.uk

This supplement supplementary information includes:

Additional methods

Figures S1 to S7

Tables S1 to S8

Additional References

Additional Methods

Soil DNA extraction

1 g of soil was mixed with 5 ml of lysis buffer pre-heated at 65 °C and 2.5 µl of 20 mg/ml Proteinase K [1]. The lysis buffer was prepared by mixing 7.1 g Na_2HPO_4, 43.8 g NaCl, 5 g CTAB, 50 ml 1 M Tris (pH 8), 100 ml 0.5 M EDTA and nanopure water to complete 500 ml. Soil slurry in lysis buffer was thoroughly mixed through the vortex and incubated for 2 hours at 65 °C with brief shaking every 15 mins to resuspend the soil. Following incubation, 2 µl of RNase A (10 mg/ml) was added to the mixture and incubated again for 45 mins at room temperature. Then, the mixture was centrifuged at 4000 x g for 10 minutes. Next, the supernatant was transferred to a fresh tube and centrifuged again at 11,000 x g for 20 minutes. The clean supernatant with minimal soil particles was transferred to a new tube, and one volume of 100% ethanol was added to the supernatant. The mixture was sequentially loaded, 700 µl at a time, onto the microfuge silica column (also known as a spin column) from the Qiagen DNeasy PowerSoil Pro Kit. The column was then centrifuged at 15,000 x g for 1 min, and the flow-through was discharged. This step was done until the entire mixture was passed through the column. The column was then treated according to the following up steps recommended by the manufacturer until DNA elution. The eluted DNA was very brown at this point. This eluted DNA will be unsuitable for PCR due to inhibition by humic acid. Therefore, the eluted DNA was washed three times following the manufacturer's protocol in the DNeasy PowerClean Pro Cleanup Kit (Qiagen) to clean the DNA further. The DNA solution was then loaded onto 0.8% agarose gel to check the integrity. DNA purity was assessed using NanoDrop® Spectrophotometer ND-1000 (Thermo Fisher Scientific, USA), and DNA was quantified following protocol for Qubit high-sensitivity dsDNA kit. Overall DNA yield was in the range of 71-1555 ng g^{-1} soil, with the lowest for MHG1 and highest for MHG9.

qPCR of 16S rRNA gene, ARGs, and MGE

The 10 µl qPCR reaction mixture contained 5 µl of 2× SensiFAST™ SYBR® No-ROX qPCR master mix (Meridian Bioscience), 0.6 µl of each primer (10 µM), 2.8 µl sterilised nuclease-free double distilled water, and 1 µl of soil DNA template. The primers used for all the genes are described in Table S3. The protocols of qPCR for different genes were different. For the 16S rRNA gene, amplification was initiated by denaturation at 95 °C for 3 min followed by 40 cycles of denaturation at 94 °C for 30 s, annealing at 50 °C for 30 s, and extension along with signal acquisition at 68 °C for 30 s. For *oqxA* and *tetX* genes, amplification was initiated by denaturation at 95 °C for 3 min followed by 40 cycles of denaturation at 95 °C for 30 s, annealing at 58 °C for 30 s, extension at 72 °C for 30 s, and signal acquisition at 83 °C for 10 s to generate the melting curve. For the rest of the genes, amplification was initiated by

denaturation at 95 °C for 3 min followed by 40 cycles of denaturation at 95 °C for 30 s, annealing at 60 °C for 30 s, extension at 72 °C for 30 s, and signal acquisition at 83 °C for 10 s.

Community profile

DNA extracted from soil was amplified with primers 341F (5'-CCTAVGGGRBCCASCAG-3') and 806R (5'-GGACTACNNGGGTATCTAAT-3'). Sequencing was done using Illumina PE250 at Novogene, UK. High-quality amplicon sequences were analysed using Qiime1 [2]. Briefly, for every sample, chimeras were removed using reference-based chimera checking following VSEARCH 2.16.0 [3]. The contigs were then clustered into OTUs using UPARSE [4]. Taxonomy was assigned using SILVA_v138 database [5].

Metagenomics

The metagenomic analysis involved two steps: quality check and assembly. Initially, the quality of the sequenced metagenomic DNA reads for each sample were assessed using FastQC version 0.11.8 [6]. Any low-quality reads (length <150 bp) were excluded from the analysis using BBDuk version38.68 [7]. This process resulted in clean, high-quality reads (>150 bp), which were then used for assembly. The processed high-quality reads were then assembled de novo into longer contiguous sequences (scaffolds) using the metaSPAdes version 3.13.1 assembler [8]. Subsequently, the annotation of ORFs into ARGs using CARD was carried out under the Perfect and Strict paradigms of RGI. The alignment was 'Perfect' when the ORFs matched completely (100%) with the curated reference sequences in CARD. The alignment was 'Strict' when the ORFs fell within the curated BLAST bit score cut-offs, allowing for variation in ORFs from the CARD reference sequence. This is particularly useful for detecting previously unknown variants of known AMR genes or altered antibiotic targets. For this study, we included both Perfect and Strict annotations. The analyses were done on a high-performance computing cluster (ADA) supported by the Research and Specialist Computing Support Service at the University of East Anglia (Norwich, UK).

Data analyses

The path diagram for the model that tests the relationships between microbial variables is shown in Figure S6 and Table S8. The model was bootstrapped 999 times to estimate the precision of the PLS parameter estimates. Path coefficients and their statistical significance (α=0.05) were reported. Measures of unidimensionality, Cronbach's alpha and DG-rho were also confirmed to be >0.7 for all the latent variables, which indicates that the block of indicators was performing well to measure their corresponding latent constructs. Loadings of each

indicator in all the latent variables were >0.7, except Shannon diversity, which indicates that the variability in the indicators was well captured by its latent construct. The quality of the path model was assessed by R^2 values for endogenous latent variables and the goodness of fit index that examines the overall prediction performance of the model.

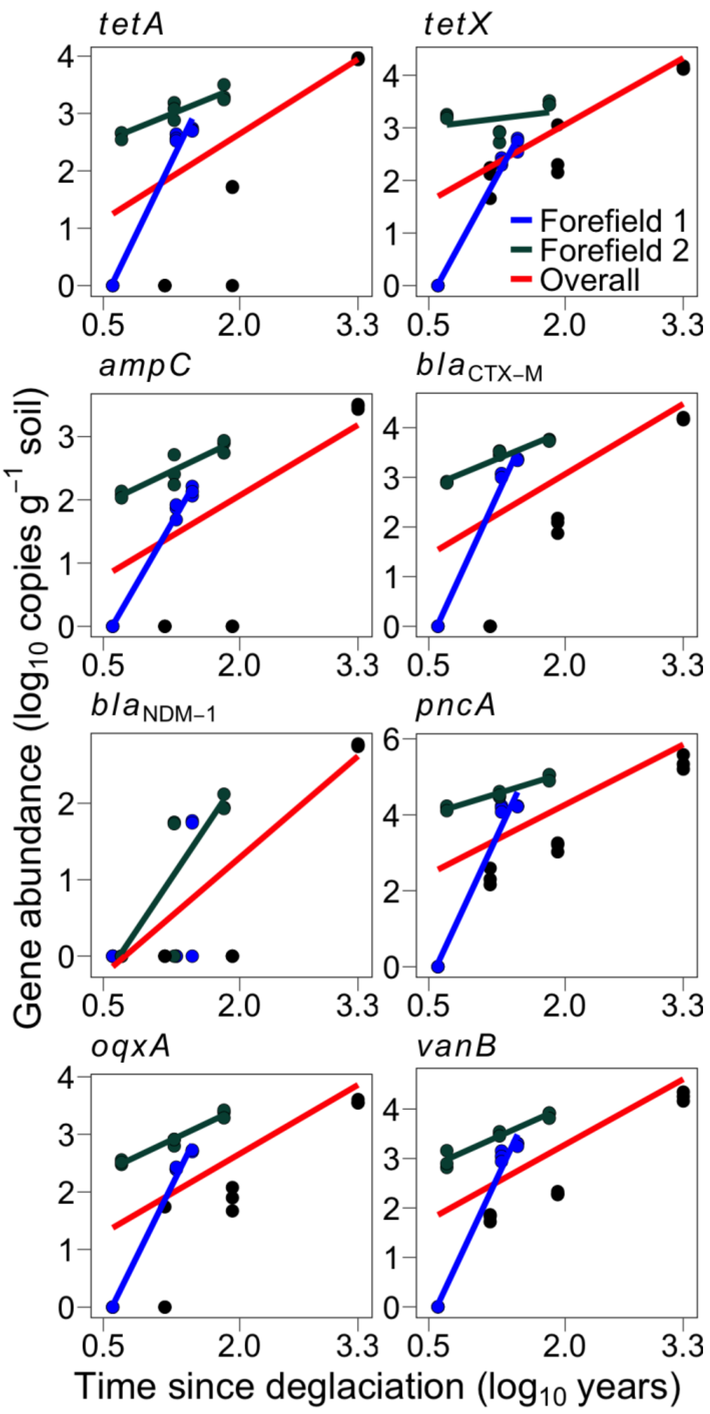

Figure S1. Relationship assessed as regression between chronosequence (time since deglaciation), represented as a continuous variable, and abundance of eight ARGs expressed as copies g^{-1} soil for two glacier forefields and overall, all samples together (red line). The associated statistics are described in Table S5. If the relationship is not significant for any of the combinations, then the regression line (coloured lines) is absent. Forefield 1 (blue line): Austre Brøggerbreen glacier forefield contains samples MHG1, MHG2, MHG3; Forefield 2 (green line): Midtre Lovénbreen glacier forefield contains samples MHG4, MHG5, MHG6.

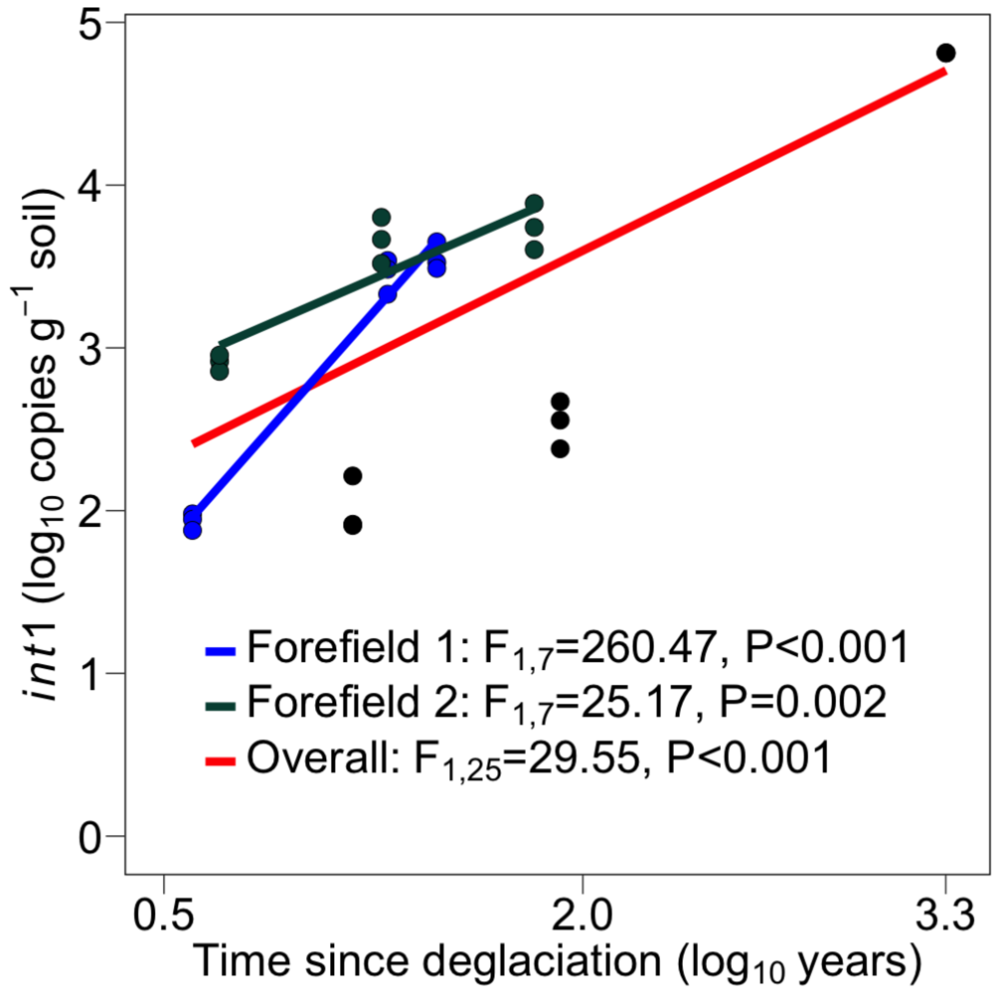

Figure S2. Relationship assessed as regression between chronosequence (time since deglaciation), represented as a continuous variable, and mobile genetic element (*int*1 abundance) for two glacier forefields and overall, all samples together (red line). Forefield 1 (blue line): Austre Brøggerbreen glacier forefield contains samples MHG1, MHG2, MHG3; Forefield 2 (green line): Midtre Lovénbreen glacier forefield contains samples MHG4, MHG5, MHG6.

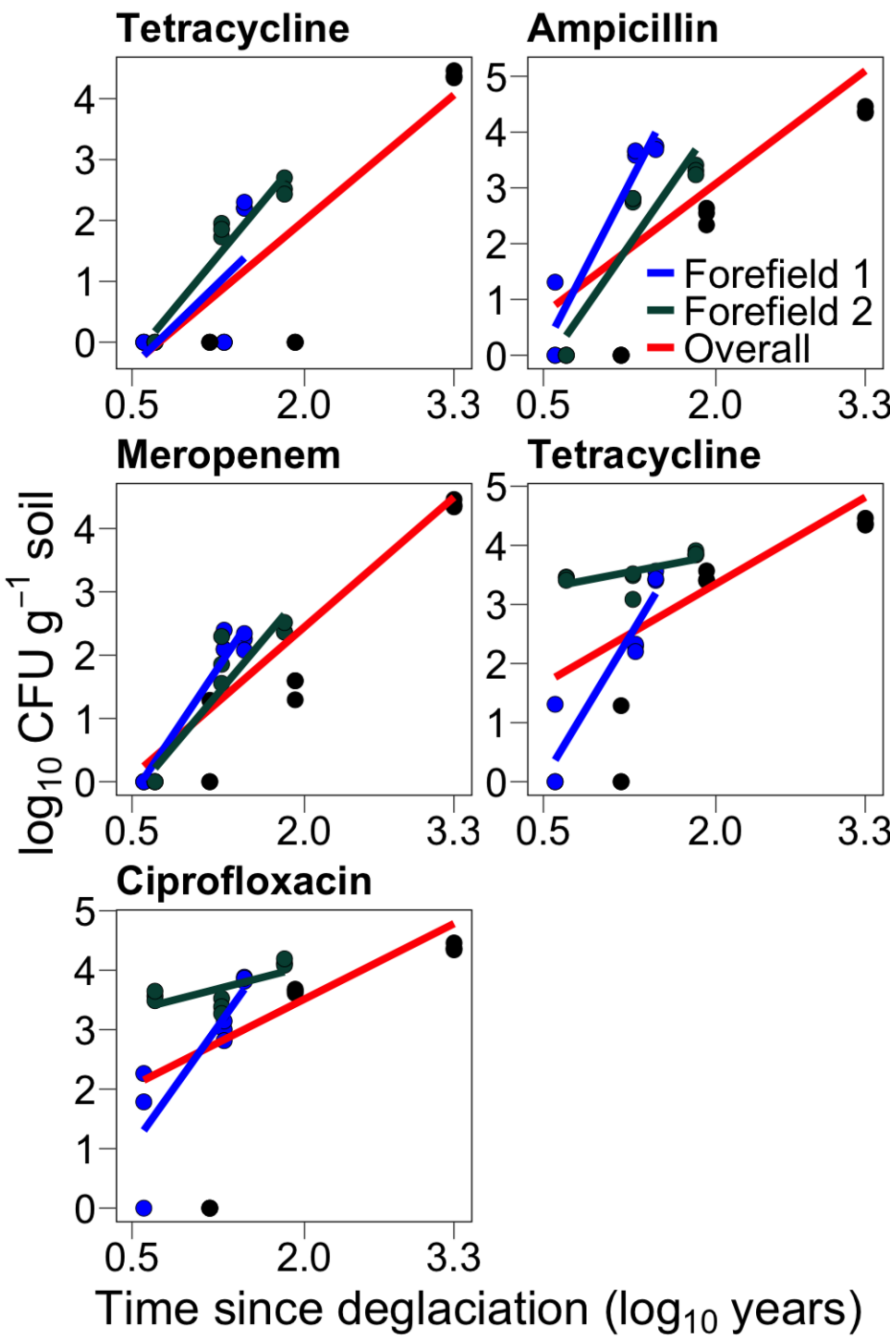

Figure S3. Relationship assessed as regression between chronosequence (time since deglaciation), represented as a continuous variable, and abundance of antibiotic resistant bacteria when exposed to five different antibiotics for two glacier forefields and overall, all samples together (red line). The associated statistics are described in Table S6. If the relationship is not significant for any of the combinations, then the regression line (coloured lines) is absent. Forefield 1 (blue line): Austre Brøggerbreen glacier forefield contains samples MHG1, MHG2, MHG3; Forefield 2 (green line): Midtre Lovénbreen glacier forefield contains samples MHG4, MHG5, MHG6.

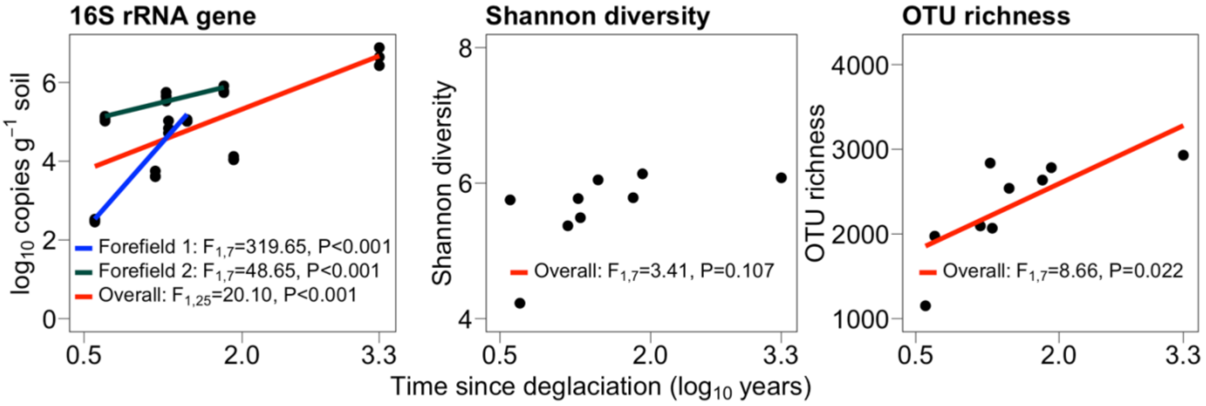

Figure S4. Relationship assessed as regression between chronosequence (time since deglaciation), represented as a continuous variable, and microbial diversity for two glacier forefields and overall, all samples together (red line). Microbial diversity is evaluated as 16S rRNA gene abundance, OTU richness and Shannon diversity. Separate linear models for different forefields were not evaluated for Shannon diversity and OTU richness. Forefield 1 (blue line): Austre Brøggerbreen glacier forefield contains samples MHG1, MHG2, MHG3; Forefield 2 (green line): Midtre Lovénbreen glacier forefield contains samples MHG4, MHG5, MHG6.

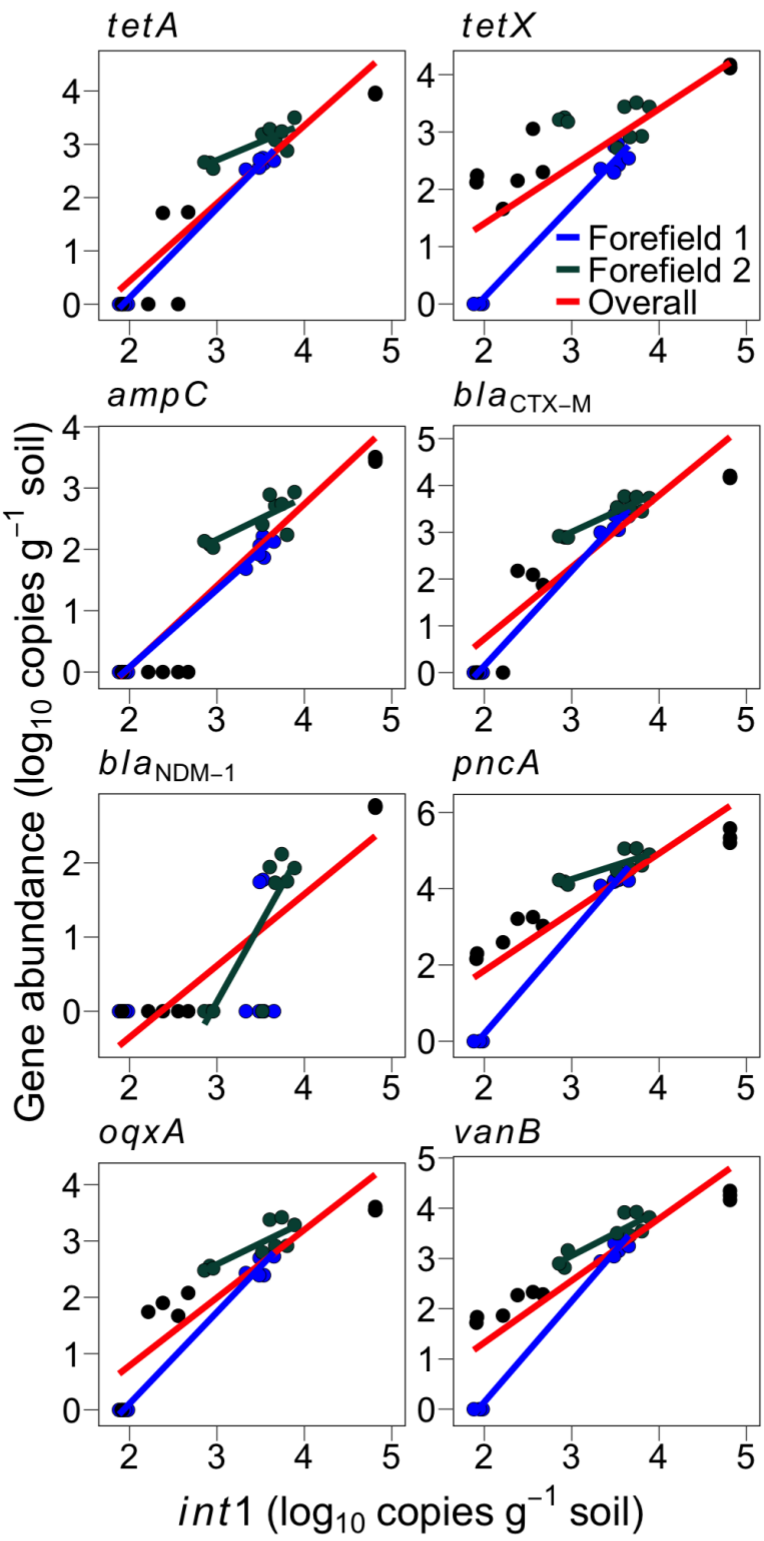

Figure S5. Relationship assessed as regression between the mobile genetic element (*int*1 abundance) and abundance of eight ARGs expressed as copies per gram soil for two glacier forefields and overall all samples together (red line). The associated statistics are described in Table S7. If the relationship is not significant for any of the combinations, then the regression line (coloured lines) is absent. Forefield 1 (blue line): Austre Brøggerbreen glacier forefield contains samples MHG1, MHG2, MHG3; Forefield 2 (green line): Midtre Lovénbreen glacier forefield contains samples MHG4, MHG5, MHG6.

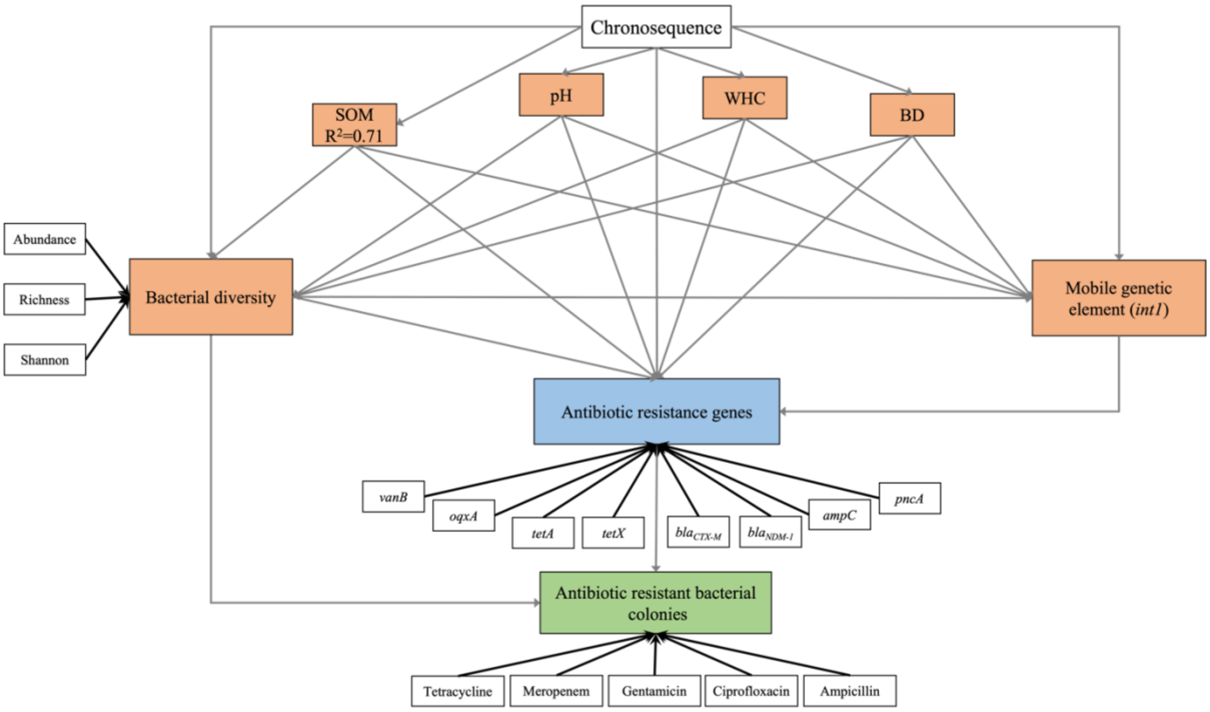

Figure S6. Partial least squares path model (PLS-PM) showing all modelled paths (Table S8).

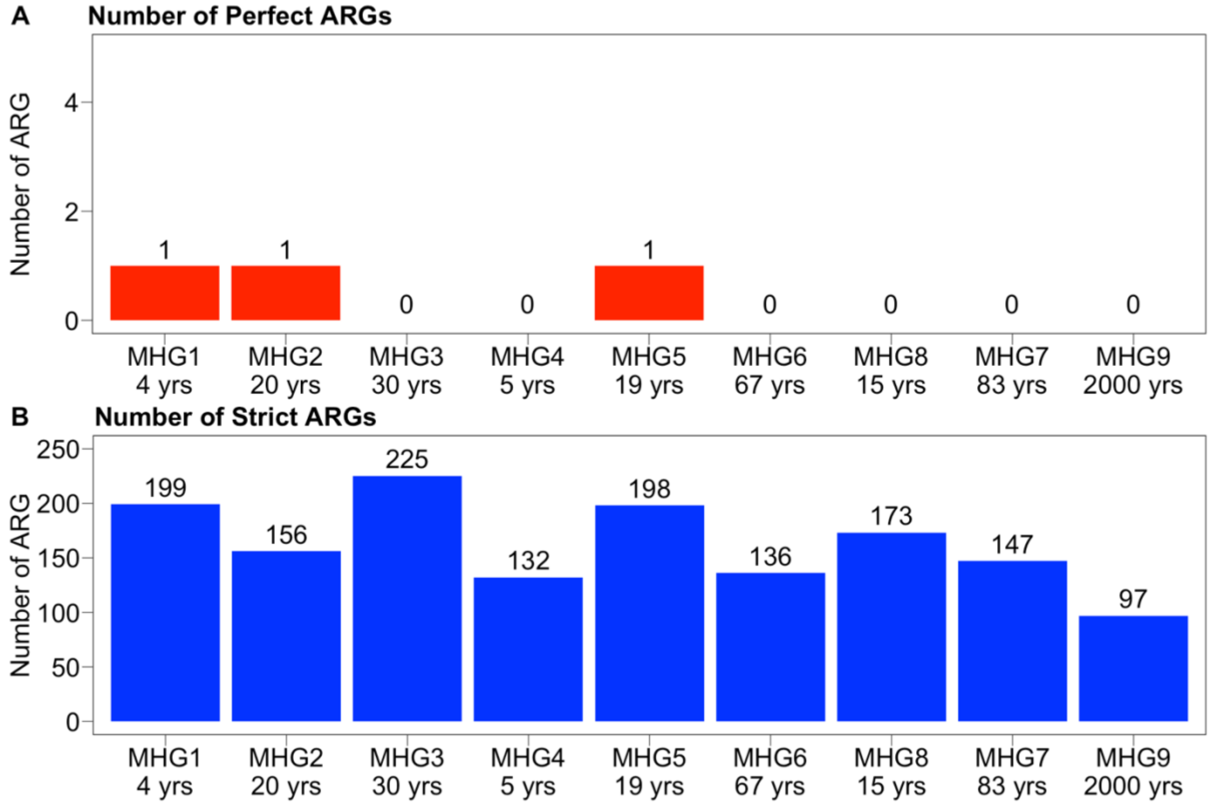

Figure S7. Number of ARGs identified from metagenomes further classified into Perfect hits (A) and Strict hits (B) along a chronosequence of deglaciated soils at different glacier forefields.

Table S1. Soil edaphic factors from sample sites in deglaciated forefields.

Sample	Time since deglaciation till sampling (years)	Distance from glacier snout (m)	Soil organic matter (SOM; %)	pH	Bulk density (BD; g cm^{-3})	Water holding capacity (WHC; %)
MHG1	4	76	0.401 ± 0.726	6.48	1.32	47.47
MHG2	20	388	0.729 ± 0.231	7.51	1.28	40.65
MHG3	30	594	1.577 ± 0.685	6.1	1.28	37.80
MHG4	5	43	1.014 ± 0.467	5.74	1.03	52.59
MHG5	19	137	1.857 ± 1.023	5.25	1.23	54.04
MHG6	67	850	2.560 ± 0.330	5.34	1.24	44.44
MHG7	83	2000 m (from Baronbreen)	0.807 ± 0.039	8	1.23	27.96
MHG8	15	180	2.635 ± 0.172	5.07	1.19	42.18
MHG9	2000	NA	48.135 ± 2.613	6.3	1.53	52.21

Table S2. Summary of antibiotic classes chosen in the study

Antibiotic class	Common antibiotics	Mechanism of action
Tetracycline	**Tetracycline**, Doxycycline, Minocycline	Broad-spectrum bacteriostatic antibiotics that bind to the 30S ribosomal subunit and prevent the aminoacyl tRNA binding to the A site of the ribosome.
Beta-lactam	Penicillin, **Ampicillin**, Amoxicillin, Cephalosporin, **Meropenem** (Carbapenems)	Bactericidal antibiotics that inhibit the synthesis of the peptidoglycan layer of bacterial cell walls. The antibiotic covalently binds to the essential penicillin-binding proteins (PBPs), which are enzymes involved in the bacterial peptidoglycan transpeptidation.
Aminoglycoside	**Gentamicin**, Streptomycin, Amikacin	Narrow-spectrum bactericidal antibiotics that inhibit peptide elongation at the 30S ribosomal subunit during protein synthesis.
Pyrazinamide	Pyrazinamide	Pyrazinamide is a widely used prodrug that prevents the growth of *Mycobacterium tuberculosis*.
Quinolone	**Ciprofloxacin**, Ofloxacin	Broad-spectrum bactericidal antibiotics that inhibit the ligase activity of the type II topoisomerases, DNA gyrase and topoisomerase IV, thereby preventing DNA replication.
Glycopeptide	Vancomycin	Multiple mechanisms. Vancomycin prevents the addition of new units to the peptidoglycan of bacterial cell walls.

Table S3. Primers for 16S rRNA gene and antibiotic-resistant genes (ARGs).

Target genes	Primer	Sequences (5′—3′)	Antibiotic class	Reference
16S rRNA	*F515*	GTGCCAGCMGCCGCGGTAA		[9]
	R806	GGACTACVSGGGTATCTAAT		
tetX	*tet*X-F	CAATAATTGGTGGTGGACCC	Tetracycline	[10]
	*tet*X-R	TTCTTACCTTGGACATCCCG		
tetB/P	*tet*B/P-F	AAAACTTATTATATTATAGTG	Tetracycline	[11]
	*tet*B/P-R	TGGAGTATCAATAATATTCAC		
tetA	*tetA*-F	CTCACCAGCCTGACCTCGAT	Tetracycline	[11]
	tetA-R	CACGTTGTTATAGAAGCCGCATAG		
ampC	*amp*-F	GCAGCACGCCCCGTAA	Ampicillin (β-lactam)	[11]
	amp-R	TGTACCCATGATGCGCGTACT		
bla_{CTX-M}	*CTX*-F	GGAGGCGTGACGGCTTTT	β-lactam	[11]
	CTX-R	TTCAGTGCGATCCAGACGAA		
bla_{IMP}	*IMP*-F	AACACGGTTTGGTGGTTCTTGTA	β-lactam	[11]
	IMP-R	GCGCTCCACAAACCAATTG		
bla_{NDM-1}	*NDM*-F	ATTAGCCGCTGCATTGAT	β-lactam	[12]
	NDM-R	CATGTCGAGATAGGAAGTG		
bla_{OXA}	*OXA*-F	CGGATGGTTTGAAGGGTTTATTAT	β-lactam	[11]
	OXA-R	TCTTGGCTTTTATGCTTGATGTTAA		
bla_{TEM}	*TEM*-F	AGCATCTTACGGATGGCATGA	β-lactam	[11]
	TEM-R	TCCTCCGATCGTTGTCAGAAGT		
AAC(3)-IIc or aacC2	*aacC2*-F	ACGGCATTCTCGATTGCTTT	Aminoglycoside	[11]

	aacC2 *R*	CCGAGCTTCACGTAAGCATTT		
pncA	*pncA*-F	GCAATCGAGGCGGTGTTC	Pyrazinamide	[13]
	pncA-R	TTGCCGCAGCCAATTCA		
intI1	*intI1*-F	CGAACGAGTGGCGGAGGGTG	Integron	[14]
	intI1-R	TACCCGAGAGCTTGGCACCCA		
tnpA-01/tnpA	*tnpA*-F	CATCATCGGACGGACAGAATT	Transposase-IS21 Group	[14]
	tnpA-R	GTCGGAGATGTGGGTGTAGAAAGT		
vanA	*vanA*-F	AAAAGGCTCTGAAAACGCAGTTAT	Vancomycin (Glycopeptide)	[11]
	vanA-R	CGGCCGTTATCTTGTAAAAACAT		
vanB	*vanB*-F	TTGTCGGCGAAGTGGATCA	Vancomycin (Glycopeptide)	[11]
	vanB-R	AGCCTTTTTCCGGCTCGTT		
oqxA	*oqxA*-F	GACAGCGTCGCACAGAATG	Quinolone, detergent, trimethoprim	[15]
	oqxA-R	GGAGACGAGGTTGGTATGGA		

Table S4. Mapping rate of the assembled reads. Mapping rate was calculated by dividing the assembled reads to the total reads [16].

Sample Id	Nr total reads	Nr of scaffolds reads (*assembled reads)*	Contigs	Rate (%)
G1	32423610	4353867	4400522	13.4
G2	24571233	3902849	3931941	15.9
G3	45385961	7401279	7486926	16.3
G4	5397955	3607618	3612825	66.8
G5	23024890	3925729	3935739	17.0
G6	28582626	5517939	5544323	19.3
G7	7436211	6065828	6070819	81.6
G8	43103842	6901841	6992399	16.0
G9	40435072	7121539	7158082	17.6

Table S5. Summary of ANOVA results evaluating the variation of individual ARGs (expressed as copies g^{-1} soil) with soil age. See Fig S1.

ARG	Overall		Forefield 1		Forefield 2	
	F-value	P-value	F-value	P-value	F-value	P-value
tetA	$F_{1,25}$=10.77	0.003	$F_{1,7}$=317.27	<0.001	$F_{1,7}$=53.34	<0.001
tetX	$F_{1,25}$=20.18	<0.001	$F_{1,7}$=744.19	<0.001	$F_{1,7}$=1.16	0.317
ampC	$F_{1,25}$= 9.23	0.006	$F_{1,7}$=832.60	<0.001	$F_{1,7}$=43.97	<0.001
bla_{CTX-M}	$F_{1,25}$=11.65	0.002	$F_{1,7}$=604.23	<0.001	$F_{1,7}$=130.38	<0.001
bla_{NDM-1}	$F_{1,25}$=28.48	<0.001	$F_{1,7}$=2.28	0.175	$F_{1,7}$=20.20	0.003
pncA	$F_{1,25}$=13.68	0.001	$F_{1,7}$=210.01	<0.001	$F_{1,7}$=162.06	<0.001
vanB	$F_{1,25}$=16.98	<0.001	$F_{1,7}$=405.94	<0.001	$F_{1,7}$=108.73	<0.001
oqxA	$F_{1,25}$=13.69	0.001	$F_{1,7}$=894.86	<0.001	$F_{1,7}$=227.75	<0.001

Table S6. Summary of ANOVA results evaluating the variation of ARB for individual antibiotics (expressed as CFU g^{-1} soil) with soil age. See Fig S3.

	Overall		Forefield 1		Forefield 2	
	F-value	P-value	F-value	P-value	F-value	P-value
Tetracycline	$F_{1,25}$=41.77	<0.001	$F_{1,7}$=5.27	0.055	$F_{1,7}$=108.57	<0.001
Ampicillin	$F_{1,25}$=27.34	<0.001	$F_{1,7}$=83.29	<0.001	$F_{1,7}$=49.74	<0.001
Meropenem	$F_{1,25}$=93.39	<0.001	$F_{1,7}$=162.56	<0.001	$F_{1,7}$=53.32	<0.001
Gentamicin	$F_{1,25}$=14.99	<0.001	$F_{1,7}$=52.66	<0.001	$F_{1,7}$=6.25	0.041
Ciprofloxacin	$F_{1,25}$= 9.72	0.005	$F_{1,7}$=21.11	0.003	$F_{1,7}$=6.79	0.035
No antibiotic	$F_{1,25}$=15.61	<0.001	$F_{1,7}$=850.61	<0.001	$F_{1,7}$=5.47	0.052

Table S7. Summary of ANOVA results evaluating the variation of individual ARGs (expressed as copies g^{-1} soil) with MGE (*int*1 abundance; copies g^{-1} soil). See Fig S5.

ARG	Overall		Forefield 1		Forefield 2	
	F-value	P-value	F-value	P-value	F-value	P-value
tetX	$F_{1,25}$=42.03	>0.001	$F_{1,7}$=348.66	>0.001	$F_{1,7}$=0.01	0.976
tetA	$F_{1,25}$=148.30	>0.001	$F_{1,7}$=959.01	>0.001	$F_{1,7}$= 1.83	0.004
ampC	$F_{1,25}$=165.78	>0.001	$F_{1,7}$=596.20	>0.001	$F_{1,7}$= 1.15	0.012
bla_{CTX-M}	$F_{1,25}$=133.71	>0.001	$F_{1,7}$=811.00	>0.001	$F_{1,7}$= 4.61	>0.001
bla_{NDM-1}	$F_{1,25}$=43.53	>0.001	$F_{1,7}$=1.17	0.315	$F_{1,7}$= 2.05	0.003
pncA	$F_{1,25}$=74.17	>0.001	$F_{1,7}$=809.67	>0.001	$F_{1,7}$= 1.47	0.006
vanB	$F_{1,25}$=96.05	>0.001	$F_{1,7}$=1044.28	>0.001	$F_{1,7}$= 2.67	0.001
oqxA	$F_{1,25}$=109.3	>0.001	$F_{1,7}$=610.44	>0.001	$F_{1,7}$= 1.62	0.005

Table S8. A-priori evidence in the literature for the modelled paths in PLS-PM.

Path	References
Chronosequence → SOM	[17, 18]
Chronosequence → pH	[19]
Chronosequence → WHC	[19]
Chronosequence → BD	[19]
Chronosequence → Bacterial diversity	[20, 21]
Chronosequence → Antibiotic resistance genes	[22]
Chronosequence → Mobile genetic element	[22]
SOM → Bacterial diversity	[20, 21]
SOM → Antibiotic resistance genes	[22]
SOM → Mobile genetic element	[22]
pH → Bacterial diversity	[23]
pH → Antibiotic resistance genes	[24, 25]
pH → Mobile genetic element	[24, 25]
WHC → Bacterial diversity	[26]
WHC → Antibiotic resistance genes	[25]
WHC → Mobile genetic element	[25]
BD → Bacterial diversity	[26]
BD → Antibiotic resistance genes	[25]
BD → Mobile genetic element	[25]
Bacterial diversity → Antibiotic resistance genes	[27]
Bacterial diversity → Mobile genetic element	[27]
Mobile genetic element → Antibiotic resistance genes	[28]
Bacterial diversity → Antibiotic resistant bacterial colonies	[27]
Antibiotic resistance genes → Antibiotic resistant bacterial colonies	[29]

Additional References

1. Marcoleta AE, Arros P, Varas MA, Costa J, Rojas-Salgado J, Berríos-Pastén C, et al. The highly diverse Antarctic Peninsula soil microbiota as a source of novel resistance genes. Sci Total Environ. 2022;810:152003.
2. Caporaso JG, Kuczynski J, Stombaugh J, Bittinger K, Bushman FD, Costello EK, et al. QIIME allows analysis of high-throughput community sequencing data. Nat Methods. 2010;7:335–6.
3. Rognes T, Flouri T, Nichols B, Quince C, Mahé F. VSEARCH: A versatile open source tool for metagenomics. PeerJ. 2016;2016:e2584.
4. Edgar RC. UPARSE: highly accurate OTU sequences from microbial amplicon reads. Nat Methods. 2013;10:996–8.
5. Quast C, Pruesse E, Yilmaz P, Gerken J, Schweer T, Yarza P, et al. The SILVA ribosomal RNA gene database project: Improved data processing and web-based tools. Nucleic Acids Res. 2013;41:D590–6.
6. Andrews S. FastQC A Quality Control tool for High Throughput Sequence Data. 2018.
7. Bushnell B, Rood J, Singer E. BBMerge – Accurate paired shotgun read merging via overlap. PLoS One. 2017;12:e0185056.
8. Nurk S, Meleshko D, Korobeynikov A, Pevzner PA. metaSPAdes: a new versatile metagenomic assembler. Genome Res. 2017;27:824–34.
9. Hernández M, Dumont MG, Yuan Q, Conrad R. Different bacterial populations associated with the roots and rhizosphere of rice incorporate plant-derived carbon. Appl Environ Microbiol. 2015;81:2244–53.
10. Fan H, Wu S, Dong W, Li X, Dong Y, Wang S, et al. Characterization of tetracycline-resistant microbiome in soil-plant systems by combination of $H_2^{18}O$-based DNA-Stable isotope probing and metagenomics. J Hazard Mater. 2021;420:126440.
11. Zhu YG, Johnson TA, Su JQ, Qiao M, Guo GX, Stedtfeld RD, et al. Diverse and abundant antibiotic resistance genes in Chinese swine farms. Proc Natl Acad Sci U S A. 2013;110:3435–40.
12. Ahammad ZS, Sreekrishnan TR, Hands CL, Knapp CW, Graham DW. Increased waterborne *bla* $_{\text{NDM-1}}$ resistance gene abundances associated with seasonal human pilgrimages to the upper Ganges river. Environ Sci Technol. 2014;48:3014–20.
13. Chen Z, Zhang W, Yang L, Stedtfeld RD, Peng A, Gu C, et al. Antibiotic resistance genes and bacterial communities in cornfield and pasture soils receiving swine and dairy manures. Environ Pollut. 2019;248:947–57.
14. Zhu Y-G, Zhao Y, Li B, Huang C-L, Zhang S-Y, Yu S, et al. Continental-scale pollution of estuaries with antibiotic resistance genes. Nat Microbiol. 2017;2:16270.
15. Wu B, Qi Q, Zhang X, Cai Y, Yu G, Lv J, et al. Dissemination of *Escherichia coli* carrying plasmid-mediated quinolone resistance (PMQR) genes from swine farms to surroundings. Sci Total Environ. 2019;665:33–40.
16. Benjamin AM, Nichols M, Burke TW, Ginsburg GS, Lucas JE. Comparing reference-based RNA-Seq mapping methods for non-human primate data. BMC Genomics. 2014;15:1–14.
17. Walker LR, Wardle DA, Bardgett RD, Clarkson BD. The use of chronosequences in studies of ecological succession and soil development. J Ecol. 2010;98:725–36.
18. Wojcik R, Eichel J, Bradley JA, Benning LG. How allogenic factors affect succession in glacier forefields. Earth Sci Rev. 2021;218:103642.
19. Delgado-Baquerizo M, Reich PB, Bardgett RD, Eldridge DJ, Lambers H, Wardle DA, et al. The influence of soil age on ecosystem structure and function across biomes. Nat Commun. 2020;11:4721.

20. Bradley JA, Arndt S, Šabacká M, Benning LG, Barker GL, Blacker JJ, et al. Microbial dynamics in a High Arctic glacier forefield: a combined field, laboratory, and modelling approach. Biogeosciences. 2016;13:5677–96.
21. Bradley JA, Singarayer JS, Anesio AM. Microbial community dynamics in the forefield of glaciers. Proceedings of the Royal Society B: Biol Sci. 2014;281:20140882.
22. Chen QL, Hu HW, Yan ZZ, Zhu YG, He JZ, Delgado-Baquerizo M. Cross-biome antibiotic resistance decays after millions of years of soil development. ISME J. 2022;16:1864–7.
23. Fierer N, Jackson RB. The diversity and biogeography of soil bacterial communities. Proc Natl Acad Sci U S A. 2006;103:626–31.
24. Han B, Ma L, Yu Q, Yang J, Su W, Hilal MG, et al. The source, fate and prospect of antibiotic resistance genes in soil: A review. Front Microbiol. 2022;13.
25. Wu J, Wang J, Li Z, Guo S, Li K, Xu P, et al. Antibiotics and antibiotic resistance genes in agricultural soils: A systematic analysis. Crit Rev Environ Sci Technol. 2023;53:847–64.
26. Chau JF, Bagtzoglou AC, Willig MR. The effect of soil texture on richness and diversity of bacterial communities. Environ Forensics. 2011;12:333–41.
27. Chen QL, An XL, Zheng BX, Gillings M, Peñuelas J, Cui L, et al. Loss of soil microbial diversity exacerbates spread of antibiotic resistance. Soil Ecol Lett. 2019;1:3–13.
28. Delgado-Baquerizo M, Hu H-W, Maestre FT, Guerra CA, Eisenhauer N, Eldridge DJ, et al. The global distribution and environmental drivers of the soil antibiotic resistome. Microbiome. 2022;10:219.
29. Jara D, Bello-Toledo H, Domínguez M, Cigarroa C, Fernández P, Vergara L, et al. Antibiotic resistance in bacterial isolates from freshwater samples in Fildes Peninsula, King George Island, Antarctica. Sci Rep. 2020;10:3145.
